# The effect and mechanism of Germacrone in ameliorating alcoholic fatty liver by inhibiting Nrf2/Rbp4

**DOI:** 10.1186/s13020-025-01132-y

**Published:** 2025-05-29

**Authors:** Ru Xiao, Jiamin Fang, Qinpo Huang, Guolin He, Xia Ou, Yang De, Shuhua Gui, Yun Zhang, Maoci Wang, Yiyuan Zhong, Dawa Zeren, Yongling Long, Changhui Liu, Tianqin Xiong

**Affiliations:** 1 School of Pharmaceutical Sciences, Guangzhou University of Chinese Medicine, No. 232 Waihuan East Road, University Town, Guangzhou, 510006 Guangdong People’s Republic of China; 2 Research Department, University of Tibetan Medicine, No. 10, Dangre Middle Road, Chengguan District, Lhasa, 850000 Tibet Autonomous Region People’s Republic of China

**Keywords:** Germacrone, Alcoholic liver disease, Oxidative stress, Nrf2, Rbp4, Lipid metabolism

## Abstract

**Background:**

Alcohol-related liver disease (ALD) is an important cause of the increase in liver disease-related morbidity and mortality worldwide. Its core pathological features are oxidative stress imbalance and lipid metabolism disorders. Nuclear factor E2-related factor 2 (Nrf2), a key regulator of oxidative stress, maintains cellular redox balance by activating antioxidant genes. However, over-activated Nrf2 may further exacerbate lipid accumulation. Retinol-binding protein 4 (Rbp4) is a key regulator of lipid metabolism, and its abnormal expression is closely related to hepatic steatosis. Therefore, regulating the balance between Nrf2 and Rbp4 may be an effective strategy to improve ALD. This study aims to explore the therapeutic effect of Germacrone on ALD and further reveal the molecular mechanism of Germacrone’s improvement of oxidative stress and lipid metabolism disorder by regulating the Nrf2/Rbp4 signaling pathway.

**Methods:**

An alcohol-induced ALD model was established in C57BL/6 mice. After continuous administration of Germacrone (21 days), the effect of Germacrone on liver lipid accumulation, oxidative stress, and pathological injury was evaluated. The core components and targets of JGST were screened by proteomics and network pharmacology, and the improvement effect of Germacrone on ALD was observed by H&E and oil red O staining, serum biochemical indices, and Western blot analysis. Subsequently, the binding of Nrf2 in the Rbp4 promoter region was analyzed by ChIP experiment. Finally, through in vivo and in vitro experiments, Nrf2 nuclear translocation and downstream target gene Rbp4 expression changes were detected, and Nrf2 knockdown or overexpression experiments were conducted to further verify its regulatory effect on Rbp4.

**Results:**

Proteomic analysis showed that the expressions of HO-1, Gsta1 and Rbp4 in the ALD model were significantly increased, and Rbp4 expression was positively correlated with liver triglyceride (TG) level. Network pharmacological predictions found that Germacrone is the core component of JGST to improve ALD. Germacrone can significantly reduce alcohol-induced liver lipid deposition, oxidative stress, and histopathological damage and significantly reduce the abnormal expression of Nuclear Nrf2 and Rbp4. ChIP experiment results showed that Nrf2 could significantly bind the Rbp4 promoter region − 1534 to − 1473 bp and transcriptionally activate its expression. Meanwhile, In vitro and in vivo experiments further verified that overexpression or activation of Nrf2 could significantly up-regulate Rbp4 expression, while knockdown or inhibition of Nrf2 could significantly decrease Rbp4 expression.

**Conclusion:**

Germacrone can protect the liver by inhibiting the Nrf2/Rbp4 signaling pathway, improving oxidative stress and lipid metabolism disorder in the ALD model. Rbp4 is a novel downstream target gene of Nrf2. As a potential drug candidate, Germacrone has great clinical application value.

**Supplementary Information:**

The online version contains supplementary material available at 10.1186/s13020-025-01132-y.

## Highlights

1. Germacrone can relieve lipid accumulation and oxidative stress in ALD.
2. It was first found that Rbp4 is the downstream target gene of Nrf2.
3. Germacrone improves alcoholic liver injury by inhibiting Nrf2/Rbp4.

**Supplementary Information:**

The online version contains supplementary material available at 10.1186/s13020-025-01132-y.

## Introduction

Alcoholic liver disease (ALD) is a significant cause of liver disease worldwide, accounting for more than 5% of global mortality, and more than half of liver disease deaths are attributed to alcohol intake [18]. The course of ALD can progress from alcoholic fatty liver to alcoholic hepatitis, liver fibrosis, cirrhosis, and even hepatocellular carcinoma. Among them, fatty liver is the early stage of ALD and is manifested by abnormal accumulation of triglycerides in the liver [6]. However, due to the lack of effective screening and clinical staging of the early stages of ALD, Most patients are diagnosed at advanced stage of the disease, resulting in difficult reversal of the disease and high mortality even after abstaining from alcohol [21]. At present, the main treatments for ALD include alcohol abstinence, corticosteroids, and adenosyl methionine, but the efficacy is limited, and there are no specific treatment drugs approved by the FDA [22]. Therefore, it is of great clinical significance to explore the molecular mechanism of ALD and develop new therapeutic targets.

Oxidative stress is one of the important pathological features of ALD, and its essence is the imbalance between free radical production and the antioxidant system [11]. Reactive oxygen species (ROS) produced during ethanol metabolism can directly or indirectly induce liver cell damage, intensify lipid accumulation, release cytokines and chemokines, and further promote liver inflammation and carcinogenesis [28]. In addition, studies have shown that oxidative stress plays an important regulatory role in fat metabolism, and high levels of ROS can promote lipid deposition by inducing lipogenesis [4, 26]. In recent years, Nrf2, as a key antioxidant transcription factor, has received much attention in the development of ALD. Overexpression of Nrf2 can increase hepatic lipid accumulation, while its knockout can significantly reduce oxidative stress-induced fat accumulation [16, 23]. In addition, RNA-Seq analysis revealed that the Nrf2/HO-1 signaling pathway was significantly activated in alcohol-induced liver injury models [27]. Therefore, moderate regulation of Nrf2 signaling pathway may be one of the key strategies to improve ALD.

Tibetan medicine has a long history, especially in the field of liver disease treatment, has accumulated rich experience. Jia-Ga-Song-Tang (JGST), also known as “SanWeiGanJiang San”, is a classic Tibetan medicine formula, mainly composed of dried ginger, white cardamom, and nutmeg. It has the effects of warming the middle and dispelling cold, strengthening the spleen and stomach, and is often used in the treatment of cold-type hepatitis and hepatomegaly [1]. In recent years, our team’s research has preliminarily revealed the mechanism of action of JGST in anti-ALD. Fang et al. [5] found that JGST can significantly improve ethanol-induced liver injury by balancing the mRNA and protein expression of HO-1 and NQO1. In addition, Chen et al. [3] pointed out that JGST can alleviate liver injury through the Nrf2/Bach1 signaling pathway, further confirming its antioxidant and anti-inflammatory effects. Recent studies have shown that JGST can also improve chronic cholestasis by activating FXR-mediated bile acid metabolism [9].

Although the above studies have revealed the potential of JGST in the treatment of liver disease, its core protein and downstream molecular targets in the ALD model have not been fully defined. This study aims to further explore the molecular mechanism of JGST in the treatment of ALD by means of proteomics and network pharmacology, focusing on its regulatory effect on the Nrf2/Rbp4 signaling pathway. In addition, we will preliminarily verify the relationship between Nrf2 and Rbp4, search for new downstream targets, and provide new scientific basis and clinical strategies for the treatment of ALD.

## Materials and methods

### Experimental animal

In this study, SPF C57BL/6 male mice were selected and purchased from Guangdong Medical Laboratory Animal Center (License number: SCXK (Guangdong) 2018-0002). Nrf2 knockout mouse (Nrf2^−/−^) was provided by Prof. Zhongqiu Liu, Guangzhou University of Chinese Medicine, and was derived from Riken Research Institute, Japan. Mice used for Nrf2 overexpression (Nrf2-AAV transfection) were purchased from Beijing Weitonglihua Laboratory Animal Technology Co., LTD. All mice were raised in the Laboratory Animal Center, College of Chinese Medicine, Guangzhou University of Chinese Medicine (License No: SYXK (Guangdong) 2019–0202). The experimental environment was strictly controlled; the temperature of the animal house was maintained at 22 ± 2 °C, the humidity was 55 ± 10%, the light cycle was 12 h of light/12 h of darkness, and the animals were free to eat standard feed and drink water. All animal experiments were conducted in accordance with the Guidelines for the Care and Use of Experimental Animals and were approved by the Experimental Animal Ethics Committee of Guangzhou University of Chinese Medicine (The Ethics number: ZYD-2022-032).

### Animal grouping and treatment

In this study, to determine the effect of JGST on Nrf2/Rbp4/HO-1 expression in ALD mice, we used the same animal grouping and drug intervention as in the previous study [5].

To explore the therapeutic effect and mechanism of Germacrone on ALD mice. After 3 days of adaptive feeding, C57BL/6 male mice were randomly divided into the following 4 groups with 6 mice in each group: (1) Control group: Mice were fed the Lieber–DeCarli standard liquid diet for 35 consecutive days without any other intervention. (2) Model group: mice were fed a liquid diet with low alcohol concentration for the first 6 days as a transition and then changed to the Lieber–DeCarli alcoholic liquid diet with 5% alcohol concentration from the 7 th day for 28 days without drug intervention. (3) Germacrone group: The feed was treated the same as the model group, and Germacrone (molecular formula: C15H22O, purity > 98%, Yuanye, Shanghai, China, 10 mg/kg) [7, 24] was given daily intragastric administration from day 8 for 21 days. (4) Brusatol group: the feed treatment was the same as the model group, and the Nrf2 antagonist Brusatol (Purity: 99.96%, Selleck, S7956, Shanghai, China, 2 mg/kg) [25] was intraperitoneally injected every other day from the 8 th day for 21 days.

In addition, in order to explore the regulatory effect of Nrf2 on Rbp4 expression. Firstly, the knockout group consisted of Nrf2 knockout mice (Nrf2^−^/^−^) and wild-type mice (WT), which were used to directly verify the effect of Nrf2 deletion on Rbp4 expression. Secondly, the drug intervention group included the Control group and the ML385-treated cell group. By inhibiting Nrf2 activity with drugs, the specific effect of Nrf2 on Rbp4 regulation was further explored. Finally, Wild-type mice (WT) and Nrf2-overexpressing mice (Nrf2 OE) were constructed in vivo animals, and similarly, normal groups and Nrf2 OE cell groups were constructed in vitro in cells to study whether Nrf2 overexpression can up-regulate the expression level of Rbp4. The construction of the ALD mouse model is the same as in previous studies [5].

On the morning of the end of the experiment, after fasting without water, blood was collected through the orbital venous plexus, centrifuged (6000×*g*, 5 min, 4 °C), and the serum was stored in − 80 °C freezer for later use. Simultaneously, mouse liver tissue was quickly extracted and divided into two parts: one for proteomic analysis and the other for pathological, biochemical, and molecular biology index detection.

### Proteomic and network pharmacological analysis

#### Proteomic analysis

According to previous studies, protein extraction was reported from the livers of mice administered by intragastric administration with ALD and JGST (JGST-L: 0.17 g/kg, JGST-M: 0.33 g/kg, and JGST-H: 0.66 g/kg) [5]. After the experiment, liver tissues were taken from mice in the Control group, Model group, and JGST intervention group, and total protein was extracted with pre-cooled RIPA lysate. The protein concentration was then quantified by the BCA method. After the experiment, samples were collected from the liver tissues of mice in the Control group, model group, and JGST intervention group, and then stored on dry ice. The proteomics experiment was entrusted to Shanghai Meggie Biotechnology Co., Ltd. for execution. Differentially expressed proteins were screened, with upregulated proteins defined as those having *P* < 0.05 and *FC* > 1.2, and downregulated proteins defined as those having *P* < 0.05 and *FC* < 0.83.

#### Network pharmacological analysis

In order to further explore the effect of JGST on ALD, the main chemical constituents of JGST and its potential targets were predicted by the Herb database (http://herb.ac.cn/) and Swiss Target Prediction database (http://www.swisstargetprediction.ch/). Through GeneCards (https://www.genecards.org/), OMIM (https://omim.org/), Disgenet database (https://disgenet.com/), Therapeutic Target Database (TDD) (http://bidd.nus.edu.sg/group/ttd/ttd.asp), and DrugBank (https://go.drugbank.com/), the disease targets associated with ALD were screened, and the common targets of JGST and ALD were obtained by intersection analysis. The common targets were imported into the STRING 11.0 database (https://string-db.org/) to construct the protein–protein interaction (PPI) network, and Cytoscape 3.7.2 software was used for network visualization analysis. The MCC algorithm in the CytoHubba plug-in was used to screen out the top 25 core targets and further combined with KEGG pathway analysis to explore the potential molecular mechanism and key targets of JGST in ALD.

### Pathological analysis

After the experiment, mouse liver tissue was taken and fixed with 4% paraformaldehyde for 12 h to preserve the structural integrity of the tissue. The fixed tissues were then dehydrated, transparent, and embedded in paraffin successively. The liver tissues were cut into continuous sections of 3 μm thickness using a paraffin microtome (Leica, Germany) and dewaxed and rehydrated according to standard procedures. Hematoxylin–Eosin (H&E) and oil red O staining were performed on the tissue sections, respectively. Five visual fields were randomly selected for each group to take microphotographs, and an Olympus optical microscope was used for observation and image recording.

### Biochemical index detection

After weighing 50 mg of mouse liver tissue, adding 10 times the volume of normal saline (1:10, w/v), homogenizing it in a low-temperature homogenizer (60 Hz, 60 s), centrifuging the homogenate at 4 °C (2500 rpm, 10 min), and taking the supernatant for biochemical index detection, including ALT, AST, CHO, TG, HDL-C, LDL-C, MDA, SOD and GSH levels. All tests were carried out in strict accordance with the kit instructions (Nanjing Jiancheng Biological Co., LTD, China). The experimental samples were repeated three times for statistical analysis.

### Cell culture

HepG2 cells were obtained from the Cell Resource Center of Peking Union Medical College. The cells were cultured in DMEM medium containing 10% fetal bovine serum and 1% penicillin–streptomycin in a 37 °C, 5% CO_2_ constant temperature incubator.

### Cell grouping and treatment

After Germacrone powder was dissolved with 0.1% DMSO, HepG2 cells were treated with different concentration gradients of Germacrone (0, 1.5625, 3.125, 6.25, 12.5, 25, 50, 100, and 200 μM) to screen the optimal concentration [14]. Then, the cells were divided into the following treatment groups: Control: the cells were not treated; Model group (Model): ethanol was used to stimulate cells. A non-cytotoxic dose is used as the dosage of medication. Germacrone low-dose group (Germacrone-L): HepG2 cells were treated with 0.5 μM Germacrone; Germacrone medium-dose group (Germacrone-M): HepG2 cells were treated with 1 μM Germacrone; Germacrone high-dose group (Germacrone-H): HepG2 cells were treated with 2 μM Germacrone; TBHQ group: HepG2 cells were treated with 50 μM TBHQ; TBHQ + Germacrone-L group: HepG2 cells were simultaneously treated with 50 μM TBHQ [13] and 0.5 μM Germacrone; TBHQ + Germacrone-M group: HepG2 cells were simultaneously treated with 50 μM TBHQ and 1 μM Germacrone; ML385 group: HepG2 cells were treated with ML385, a 5 μM Nrf2 inhibitor [12]. After the cells in each group were treated for 24 h, 200 mM ethanol was added to stimulate the cells for 2 h to observe the degree of cell damage, and cell samples were collected for subsequent analysis.

In addition, for Nrf2 shRNA (shNrf2) and Nrf2 overexpression vector (Nrf2 OE) were designed, synthesized, and validated by TsingKe (Shanghai, China). Virus transfection was performed according to the instructions. Cells were spread evenly in six-well plates and transacted when cells grew to 50–60% confluence for 24 h. The transfection solution was then removed and screened with puromycin, the fresh medium was replaced for follow-up experiments.

### Cell activity assay

HepG2 cells were inoculated into 96-well plates with a density of 1 × 10^4^ cells/well. After cell adhesion, 10 μL of CCK8 reagent (FD3788, APExBIO, Hangzhou, China) was added to each well according to the above classification and treatment. The light absorption value was measured at 450 nm with a multifunctional enzymograph, and the percentage of cell activity was calculated.

### Immunofluorescence assay

HepG2 cells were evenly spread on a slide culture plate. After treatment, the cells were fixed with 4% paraformaldehyde for 20 min and washed with PBS three times for 5 min each time. Subsequently, 1× membrane rupture working solution was added and incubated at room temperature for 20 min, and after washing with PBS, 10% goat serum was used for 30 min to reduce non-specific binding. After closure, anti-Nrf2 antibody was added to the wet box and incubated at 4 °C overnight. The next day, wash with PBS 3 times, 5 min each time, and add the corresponding fluorescent secondary antibody (SA00013-4, Proteintech, Wuhan, China), and incubate at 37 °C for 1 h. After washing, the slides were sealed with DAPI-containing mounting medium and observed under a fluorescence microscope (Olympus, Japan) as soon as possible. Fluorescence images of the cells were obtained, and quantitative analysis was performed using ImageJ software.

### Chromatin immunoprecipitation (ChIP)

HepG2 cells were treated with Germacrone and ethanol, fixed with 1% paraformaldehyde solution for 10 min, and 10× glycine solution was added to terminate the crosslinking reaction. After cell lysis, nuclear chromatin was interrupted by ultrasonic fragmentation, and fragments of about 200–1000 bp in length were obtained. The chromatin lysate was mixed with CHIP-grade Protein G magnetic beads for immunoprecipitation by adding specific Nrf2 antibody (12721S, CST, USA) and incubated overnight at 4 °C. After incubation, the magnetic beads were washed, chromatin fragments were released, and decrosslinked buffer was added for decrosslinking. DNA fragments were purified by centrifugal column. Finally, RT-qPCR was used to analyze the enrichment efficiency of specific target gene promoter regions to verify the binding ability of Nrf2 to its downstream target genes.

### Western blot assay

In order to detect the expression levels of Nrf2 and its downstream signaling pathway-related proteins, the cytological and plasma protein extraction kit (P0028, Beyotime, Shanghai, China) was used to lyse tissues or cells and extract total protein. The PVDF membrane with samples was incubated at 4 °C with primary antibody overnight. Primary antibodies used include Nrf2 antibody (AF0639, Affinity, China), LaminB antibody (AF5161, Affinity, China), HO-1 antibody (ab13243, Abcam, Cambridge, MA, USA), Rbp4 antibody (ab109193, Abcam, Cambridge, MA, USA), and β-actin antibody (AF7018, Affinity, Changzhou, China). The next day, rabbit secondary antibody (SA000012, Proteintech, Wuhan, China) (1:10,000) labeled with HRP was incubated at 25 °C for 1 h. The ECL chemiluminescence kit was used to visualize the PVDF membrane in the GEL imaging system (Bio-Rad, USA).

### RNA extraction and RT-qPCR detection

Total RNA was extracted from cells or tissues using TRIzol reagent (Invitrogen, USA), and RNA concentration and purity were determined by NanoDrop spectrophotometer. The extracted RNA was reverse-transcribed into cDNA using a cDNA Synthesis Kit (Kelanase Biotechnology Co. Ltd., Guangzhou, China). RT-qPCR assay was performed on ABI StepOnePlus real-time fluorescence quantitative PCR system using 2× SYBR Green qPCR Premix (Takara, Japan) to analyze the mRNA expression levels of Nrf2 and its downstream target genes. The primer sequence as shown in Table 1. Data were standardized using the 2^−ΔΔCt^ method, and the results were calibrated using GAPDH as the internal reference.

**Table 1:** Primers for RT-qPCR

RNA	Sequences (5ʹ to 3ʹ)
HO-1	5ʹ-AAGAGGCCAAGACTGCGTTC-3 (forward) 5ʹ-CATTGGTGCTGCTGATGACG-3ʹ (reverse)
NQO1	5ʹ-GAAGACATCATTCAACTACGCC-3ʹ (forward) 5ʹ-GAGATGACTCGGAAGGATACTG-3ʹ (reverse)
Nrf2	5ʹ-TCCAAGTCCAGAAGCCAAACTGAC-3ʹ (forward) 5ʹ-GGAGAGGATGCTGCTGAAGGAATC-3ʹ (reverse)
Rbp4	5ʹ-ACTCCTGCCGCCTCCTGAAC-3ʹ (forward) 5ʹ-TCCTCCTGCCGCTGCCTTAC-3ʹ (reverse)
FASN	5ʹ-GTGGTGGGCTTGGTGAACTGTC-3ʹ (forward) 5ʹ-GTGGTGGGCTTGGTGAACTGTC-3ʹ (reverse)
SCD1	5ʹ-CCACCACCACCACCATTACAGC-3ʹ (forward) 5ʹ-AGTAGAGGGGCATCGTCTCCAAC-3ʹ (reverse)
SREBP1	5ʹ-GCTGTTGGTGCTCGTCTCCTTG-3ʹ (forward) 5ʹ-GCTTGCGATGCCTCCAGAAGTAC-3ʹ (reverse)
GAPDH	5ʹ-CAGGAGGCATTGCTGATGAT-3ʹ (forward) 5ʹ-GAAGGCTGGGGCTCATTT-3ʹ (reverse)

### Statistical analysis

All data were statistically analyzed using SPSS 21.0 software, and the results were expressed as mean ± standard deviation. After conducting normality tests (Kolmogorov–Smirnov test) and homogeneity of variance tests (Bartlett's test), data conforming to a normal distribution were analyzed by one-way ANOVA. For datasets with homogeneous variance, pairwise comparisons were performed using the LSD method, while Dunnett’s T3 test was applied for datasets with heterogeneous variance. Results were expressed as mean ± standard error (SE). Pearson correlation analysis was used to calculate correlation coefficients for normally distributed data.

## Result

### Proteomic analysis revealed potential targets for JGST to improve ALD

**Fig. 1:**
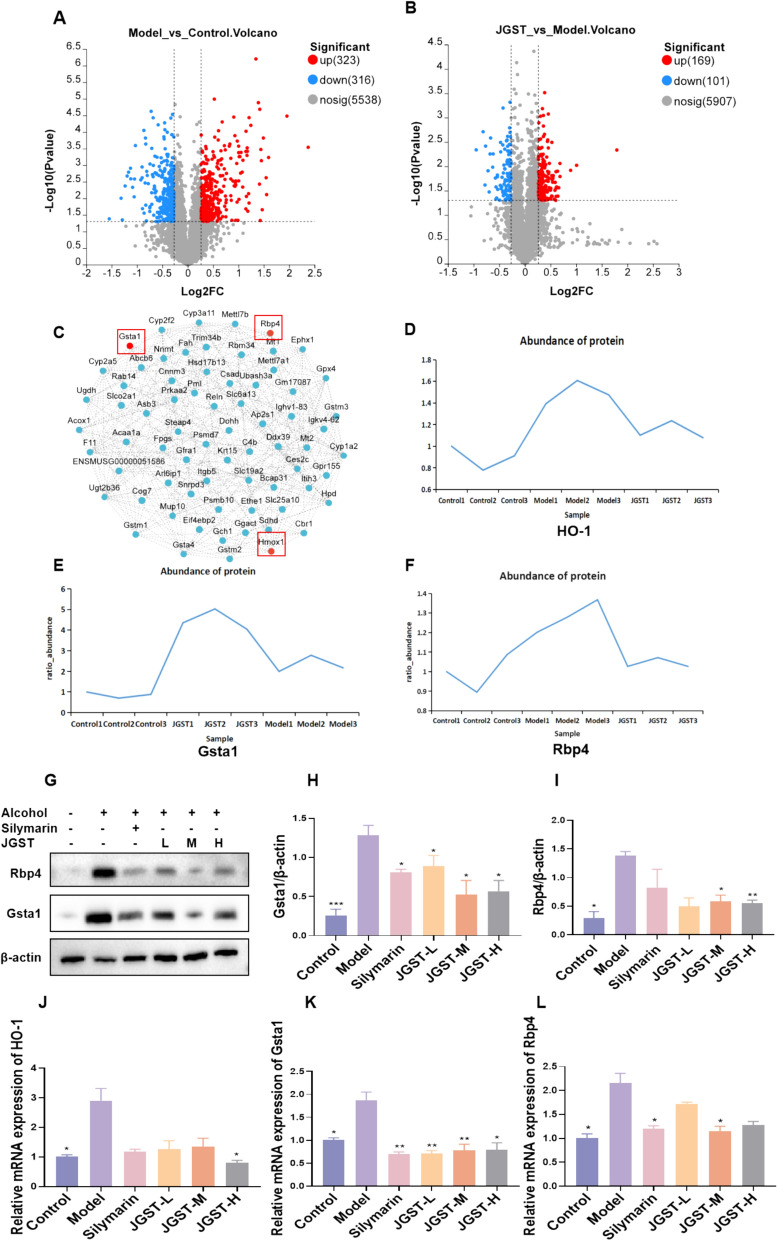
Proteomic analysis of JGST improving ALD. **A** Volcanic diagram shows differentially expressed proteins between the ALD model group and the Control group (n = 3); **B** the volcano plot displays the differentially expressed proteins between the JGST intervention group and the ALD model group, with red dots representing upregulated proteins, green dots representing downregulated proteins, and gray dots representing proteins with no significant differences; **C** visualization of differential proteins; **D**–**F** protein expression levels of HO-1, Gsta1, and Rbp4 in proteomics; **G**–**I** western blot was used to detect the protein expression levels of Rbp4 and Gsta1 (n = 3); **J**–**L** RT qPCR was used to detect the mRNA expression levels of HO-1, Gsta1, and Rbp4 (n = 6). Compared with the model group, *P < 0.05, ** P < 0.01, ***P < 0.001

Previous studies have shown that JGST can effectively improve liver and intestinal damage in mice with alcoholic liver disease [5]. In order to further investigate the underlying molecular mechanism, proteomic analysis of mouse liver tissues was performed. Through mass spectrometry, 6177 proteins were successfully identified, the length of peptide segments ranged between 5–20 amino acids, and the error distribution was within 20 ppm, these parameters all met the quality control requirements of mass spectrometry (Supplementary Fig. 1 A–C). Proteomic analysis showed that compared with the Control group, there were 639 differentially expressed proteins in the liver of the model group, among which 323 proteins were upregulated and 316 proteins were downregulated. Compared with the model group, a total of 270 differential proteins were detected in the JGST intervention group, of which 169 proteins were upregulated and 101 proteins were downregulated (Fig. 1A, B). These results suggest that JGST can protect against ethanol-induced liver injury through multi-target regulatory mechanisms.

The intersection analysis of differential proteins in the Control group, Model group, and JGST intervention group was carried out, and 88 common key differential proteins were obtained. Through PPI network Visualization analysis, it was found that the liver protective effect of JGST may be closely related to HO-1, Gsta1, and Rbp4 (Fig. 1C–F). Further validation analysis of the above differential proteins showed that the protein and mRNA expression levels of HO-1, Gsta1, and Rbp4 were significantly increased in ALD mice. After the intervention of low, medium, and high doses of JGST, the expression of these proteins showed a significant inhibition trend (Fig. 1G–L).

The above results were consistent with the trend of TMT proteomic analysis, suggesting that JGST may alleviate ethanol-induced liver injury by regulating the expression of Rbp4 and Nrf2 target proteins (such as HO-1 and Gsta1). These results further verified the protective effect of JGST in ALD.

### Network pharmacological analysis revealed the key role of Germacrone in anti-ALD of JGST

In order to further explore the key components and targets of JGST in improving ALD, this study combined network pharmacological methods with systematic analysis. Firstly, 1191 JGST-related drug targets were screened through the Herb database and Swiss Target Prediction database. At the same time, 1306 ALD-related genes were obtained by different disease databases. The intersection analysis of the two results yielded a total of 228 common targets, which may be potential targets for JGST to improve ALD (Supplementary Fig. 2 A). Following PPI and Cytoscape visualization analysis of all the targets (Supplementary Fig. 2B), the top 25 core targets were selected, which have high network importance and may play a key role in JGST's treatment of ALD (Supplementary Fig. 2 C). Meanwhile, KEGG enrichment analysis revealed that the overlapping targets between ALD and JGST were significantly enriched in lipid metabolism pathways, such as “Regulation of lipolysis in adipocytes” and “Linoleic acid metabolism.” The former pathway alleviates hepatic lipid deposition by modulating lipolytic balance, while the latter may mediate inflammation and oxidative stress (Supplementary Fig. 3). These findings suggest that JGST might ameliorate the progression of liver injury by intervening in lipid metabolism disorders, a mechanism that is consistent with the role of lipid-regulatory proteins identified in proteomic studies in disease development.

Further corresponding core targets to JGST components one by one, the results showed that Germacrone corresponded to 15 core targets, covering the largest number of targets (Supplementary Tables 1 and 2), this suggesting that Germacrone is a key component in JGST for playing an anti-ALD role.

### Germacrone improves lipid accumulation and oxidative stress in mouse models of ALD

**Fig. 2:**
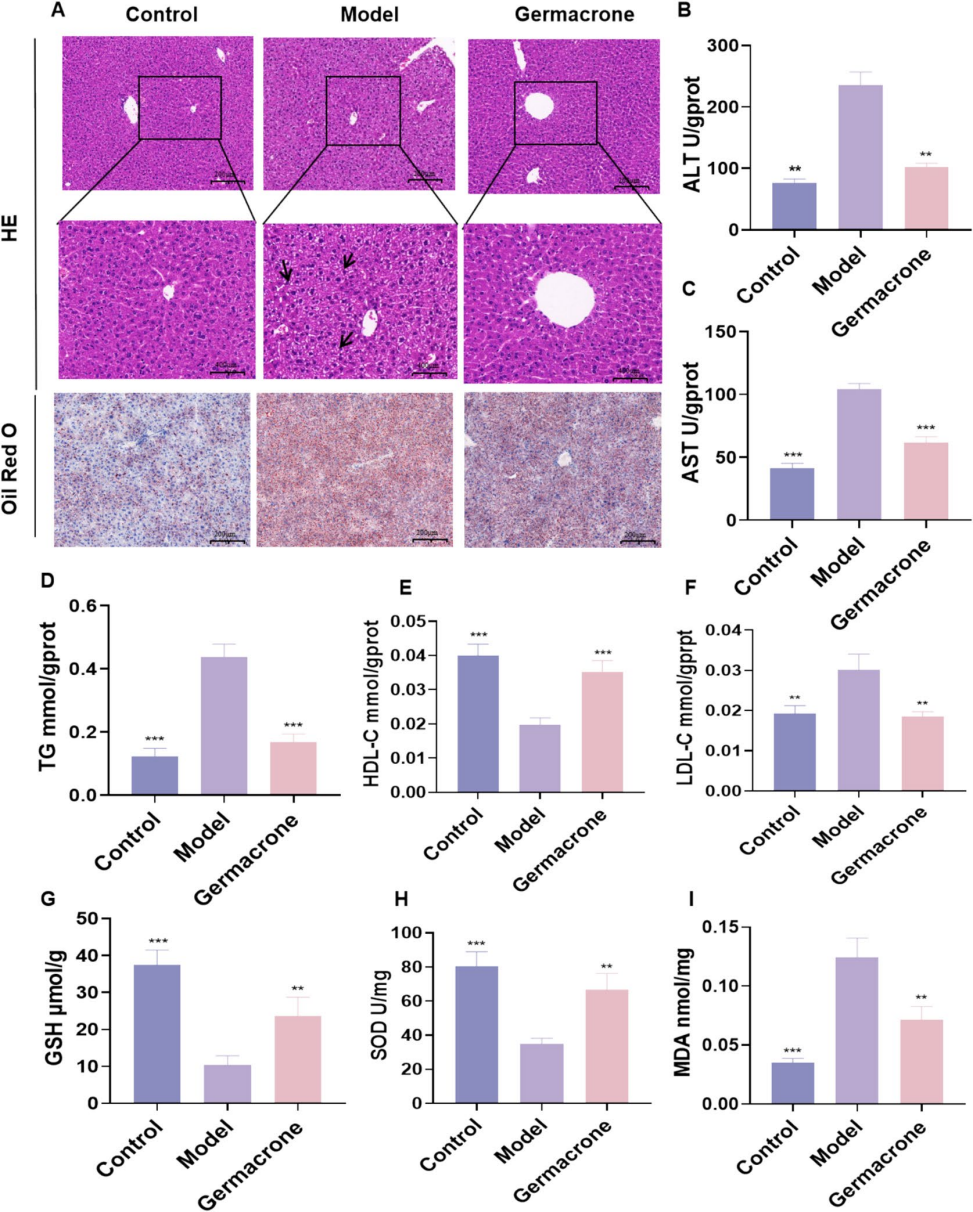
Germacrone improves lipid accumulation and oxidative stress in a mouse model of ALD. **A** H&E staining revealed pathological changes in the mouse liver; Oil red O staining showed lipid accumulation in hepatocytes. **B**–**I** biochemical kit was used to detect ALT, AST, TG, HDL-C, LDL-C, GSH, SOD, and MDA levels in liver tissues (n = 6). Compared with the model group, *P < 0.05, **P < 0.01, ***P < 0.001

To validate the network pharmacology hypothesis, this study further investigated the effect of Germacrone on the ALD mouse model H&E staining showed that there were obvious fat vacuoles at the edge of the liver in the model group, and the structure of hepatocytes was disordered, accompanied by bridging necrosis. After Germacrone intervention, liver fat vacuoles were significantly reduced, and liver cell morphology tended to be normal. Oil red O staining further confirmed that lipid accumulation in hepatocytes in the mice model group was significantly increased, and Germacrone treatment significantly improved this phenomenon (Figs. 2A). Liver biochemical parameters showed that Germacrone intervention significantly reduced ALT and AST levels, reduced the abnormal accumulation of TG, increased HDL-C content, and decreased the LDL-C level in the model group (Figs. 2B–F). In addition, Germacrone significantly reversed the increase in MDA, restored GSH and SOD levels in the model group (Fig. 2G–I), suggesting that Germacrone can play a protective role in the liver of ALD mice by improving oxidative stress and lipid metabolism disorders.

### Germacrone improves alcohol-induced lipid accumulation and oxidative stress in HepG2 cells

**Fig. 3:**
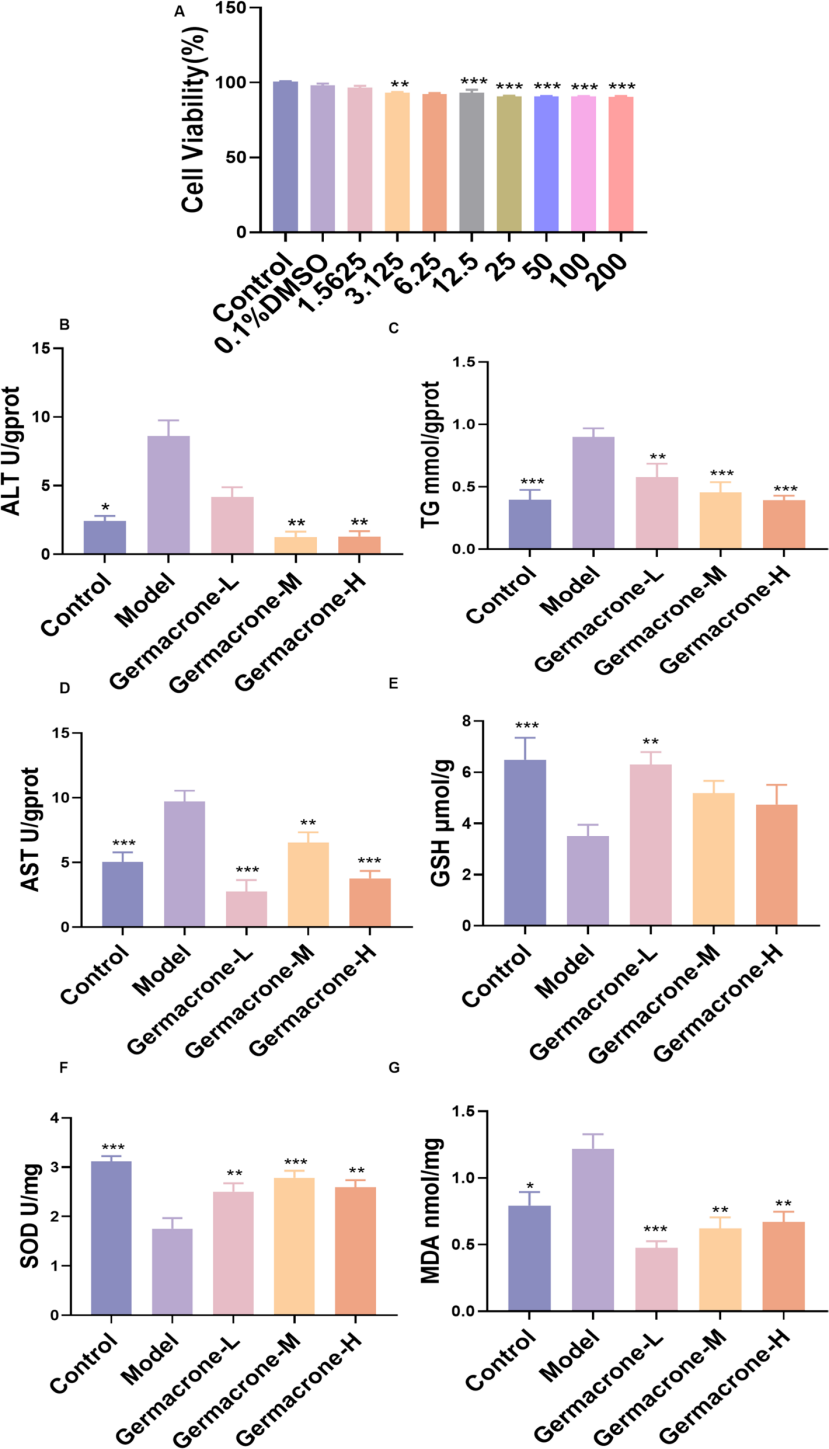
Germacrone improves alcohol-induced lipid accumulation and oxidative stress in HepG2 cells. **A** The effect of different concentration gradients of Germacrone on HepG2 cell activity was detected by CCK-8 assay (n = 5); **B**–**G** A biochemical kit was used to detect the levels of ALT, AST, TG, GSH, SOD, and MDA in the Control group, Model group, Germacrone-L group, Germacrone-M group, and Germacrone-H group (n = 4–6). Compared with the Model group, *P < 0.05, **P < 0.01, ***P < 0.001

To further verify the mechanism of action of Germacrone, the protective effect of Germacrone on alcohol-induced hepatocyte injury was investigated in a cell model. First, the non-toxic concentration range of Germacrone on HepG2 cells was determined by the CCK-8 method. The results showed that the Germacrone solution with concentrations of 0.5, 1.0, and 2.0 μM had no significant effect on cell activity, so the intervention concentration was selected for subsequent experiments (Fig. 3A). In alcohol-induced HepG2 cell models, administration of Germacrone significantly reduced intracellular ALT and AST levels and reduced abnormal accumulation of TG and MDA (Fig. 3B–D, G). At the same time, Germacrone intervention significantly increased the contents of intracellular SOD and GSH (Fig. 3E, F), effectively alleviating oxidative stress and lipid metabolism disorders. These results indicate that Germacrone has significant antioxidant and lipid-regulating effects in both in vivo and in vitro models, further confirming its central role as the key component of JGST in improving ALD.

### Germacrone inhibits abnormal expression of the Nrf2 signaling pathway and its downstream target genes

**Fig. 4:**
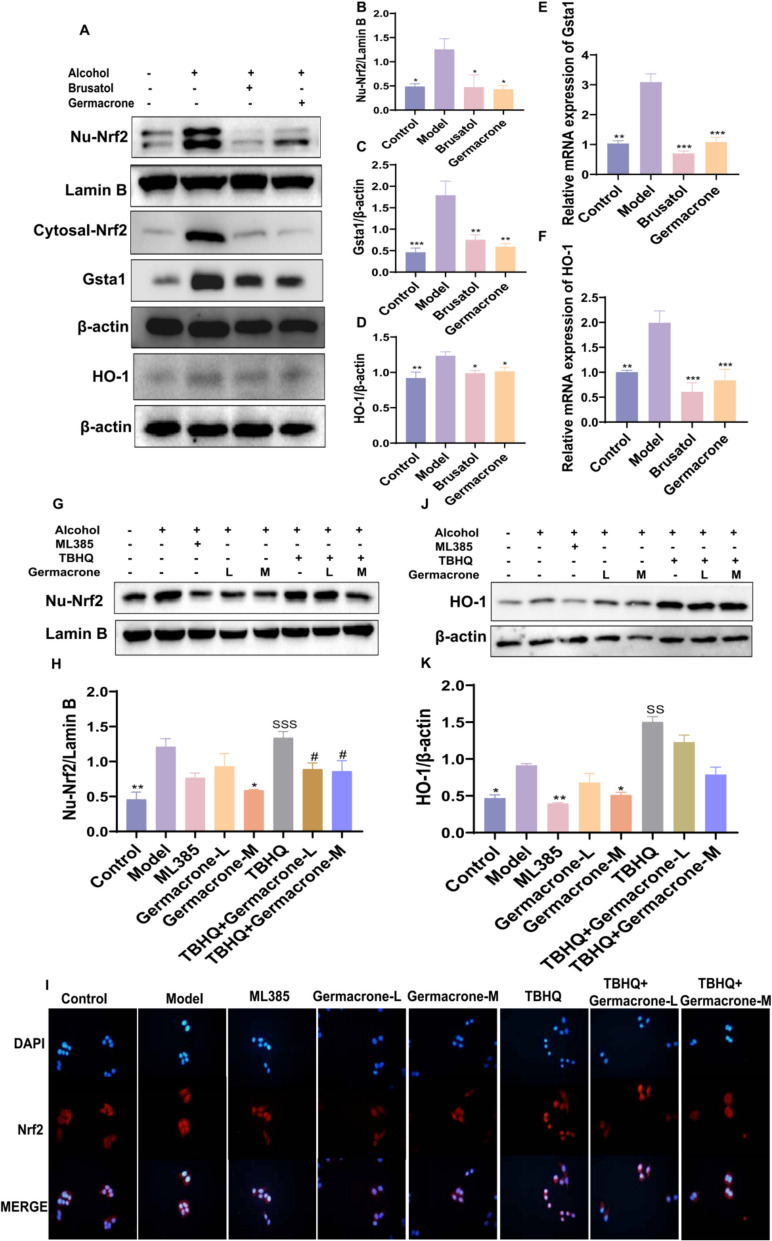
Germacrone regulates Nrf2 nuclear translocation and downstream target gene expression in ALD. **A**–**D** Western blot analysis of Nrf2, HO-1, and Gsta1 protein expression levels in liver tissues of the Control group, Model group, Brusatol group and Germacrone group (n = 3). **E**, **F** The mRNA levels of HO-1 and Gsta1 in liver tissues of the Control group, Model group, Brusatol group, and Germacrone group were detected by RT-PCR (n = 6). **G**, **H** Western blot analysis showed the expression level of Nrf2 protein in each group (n = 3). **I** Immunofluorescence was used to observe the localization and expression of Nrf2 in HepG2 cells (magnification 200×, n = 3). **J**, **K** Western blot analysis showed HO-1 protein expression levels under different treatments (n = 3). Compared with the model group, *P < 0.05, **P < 0.01, ***P < 0.001; #P < 0.05, ##P < 0.01, ###P < 0.001 compared with the TBHQ group

Nrf2 is a highly conserved alkaline leucine zipper transcription factor. Under oxidative stress conditions, Nrf2 is released from the Keap1 complex, translocated into the nucleus, and activates the expression of downstream antioxidant stress genes such as HO-1, Gsta1, and NQO1 [2]. To investigate the regulatory effect of Germacrone on the Nrf2 signaling pathway and its specific mechanism in the ALD model, we first validated it in animal model. Western blot and RT-qPCR results showed that compared with the control group, the nuclear translocation of Nrf2 was significantly increased in the liver tissue of the model group mice, while the protein and mRNA expression levels of downstream target genes HO-1 and Gsta1 were significantly increased. After Germacrone intervention, these abnormal expressions were significantly downregulated, exhibiting inhibitory effects similar to those of the Nrf2-specific inhibitor Brusatol (Figs. 4A–F).

The above findings were further validated in cell experiments. Through immunofluorescence and Western blot analysis, we observed a significant increase in Nrf2 expression in HepG2 cell nuclei under alcohol stimulation, showing a trend similar to that of the Nrf2 agonist TBHQ treatment group. However, treatment with Germacrone significantly inhibited nuclear translocation of Nrf2, indicating that Germacrone effectively regulated the oxidative balance of liver cells (Fig. 4G–I). In addition, compared with the Control group, the expression of HO-1 protein was upregulated in the model group, and the effect of abnormal HO-1 expression was significantly reduced after Germacrone treatment, but the effect of TBHQ in reversing the abnormal HO-1 expression was not observed (Fig. 4J, K). In summary, Germacrone can significantly improve the oxidative stress imbalance in the ALD model by inhibiting the nuclear translocation of Nrf2 and the abnormal expression of downstream target genes HO-1, Gsta1, and NQO1.

### Germacrone inhibits Rbp4 expression through Nrf2 and improves lipid metabolism

**Fig. 5:**
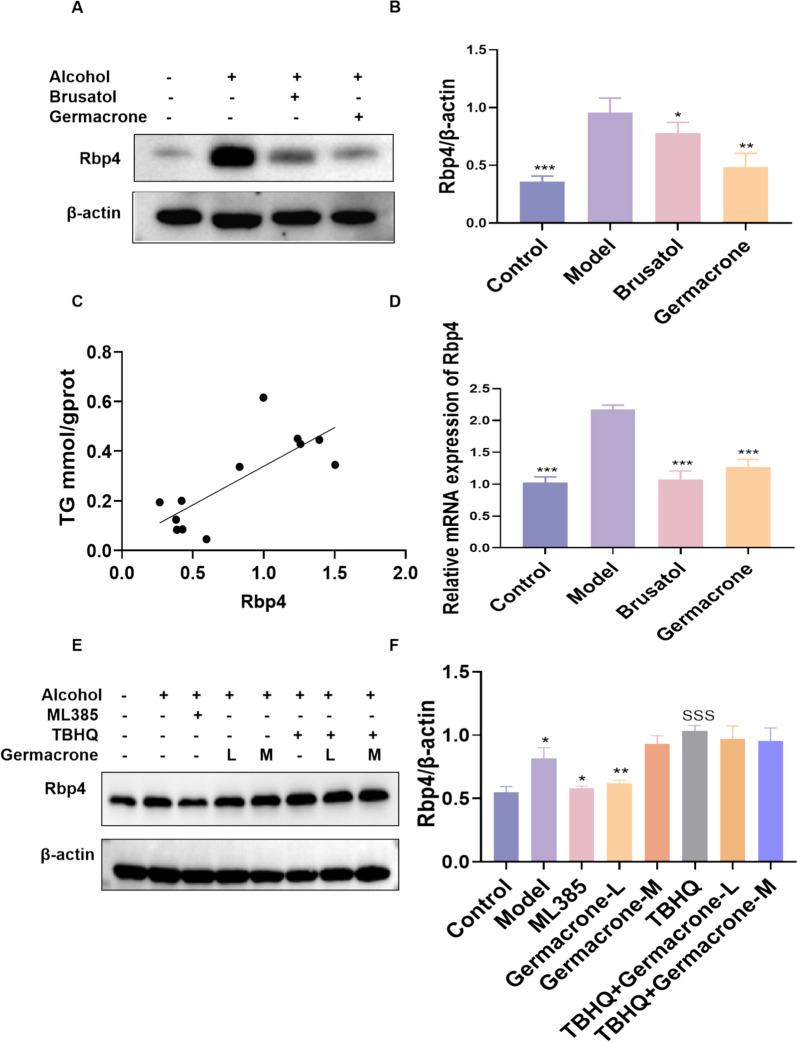
Germacrone inhibits Nrf2-dependent Rbp4 expression in the ALD model. **A**, **B** The protein expression levels of Rbp4 in the ALD model treated with Germacrone and Brusatol (n = 3). **C** Correlation analysis of liver TG content and Rbp4 expression. **D** Rbp4 mRNA expression in HepG2 cells in the Control group, Model group, Brusatol group, and Germacrone group was analyzed by RT-qPCR (n = 6). **E**, **F** Western blot was used to detect the expression level of Rbp4 protein under different conditions (n = 3). Compared with the model group, *P < 0.05, **P < 0.01, ***P < 0.001

Exacerbation of oxidative stress and disturbance of lipid metabolism are important factors in the occurrence of ALD. Rbp4 is an important regulatory protein of lipid metabolism, and its abnormal expression has been shown to be closely related to oxidative stress and lipid accumulation [20, 29]. However, there is no direct evidence for a relationship between Rbp4 and Nrf2. Therefore, in order to verify the regulatory effect of Germacrone on lipid metabolism, this study examined the expression level of Rbp4 and its relationship with the Nrf2 signaling pathway. Western blot and RT-PCR results revealed that the expression level of Rbp4 in the ALD mouse model was significantly increased due to alcohol stimulation, and correlation analysis showed that the expression level of Rbp4 was significantly positively correlated with liver TG content (Fig. 5A–C). Furthermore, we examined the expression of lipogenesis-related genes and detected that although FASN, SREBP1, and SCD showed an upward trend in the ethanol-exposed group, and a downward trend was observed following Germacrone intervention, none of these differences reached the threshold of statistical significance. Based on these findings, Rbp4, which demonstrated a significant linear correlation with lipid accumulation, was ultimately selected as the primary research target to further investigate its molecular regulatory mechanisms in alcohol-induced liver injury (Supplementary Fig. 4 A–C). After treating with Germacrone, the protein and mRNA expression of Rbp4 were significantly down-regulated, showing similar effects to those of the Nrf2 inhibitor Brusatol (Fig. 5D).

In cell model, we further observed that Germacrone significantly inhibited the abnormal expression of Rbp4 in HepG2 cells. Notably, the expression level of Rbp4 also decreased significantly after treatment with the Nrf2 antagonist ML385, while the expression level of Rbp4 increased significantly in the Nrf2 agonist TBHQ treatment group, which was consistent with the trend in the model group (Figs. 5E, F). This suggests that the abnormal expression of Rbp4 may be regulated by the Nrf2 signaling pathway. Germacrone can reduce the lipid accumulation and metabolic disorders in hepatocytes by inhibiting the Nrf2-dependent expression of Rbp4.

In conclusion, Germacrone can play a protective role against alcohol-induced liver cell injury by inhibiting Nrf2 and down-regulating the expression of Rbp4, and at the same time, it reveals the potential relationship between the Nrf2 signaling pathway and lipid metabolism.

### Nrf2 transcription regulates the expression of Rbp4

In order to further clarify the molecular mechanism of Nrf2 regulating Rbp4 expression, this study employed a comprehensive strategy integrating in vivo and in vitro reciprocal validation approaches combined with ChIP-seq and ChIP-qPCR techniques. The results demonstrated that Nrf2 directly binds to the promoter region of the Rbp4 gene, thereby establishing the regulatory relationship between them.

**Fig. 6:**
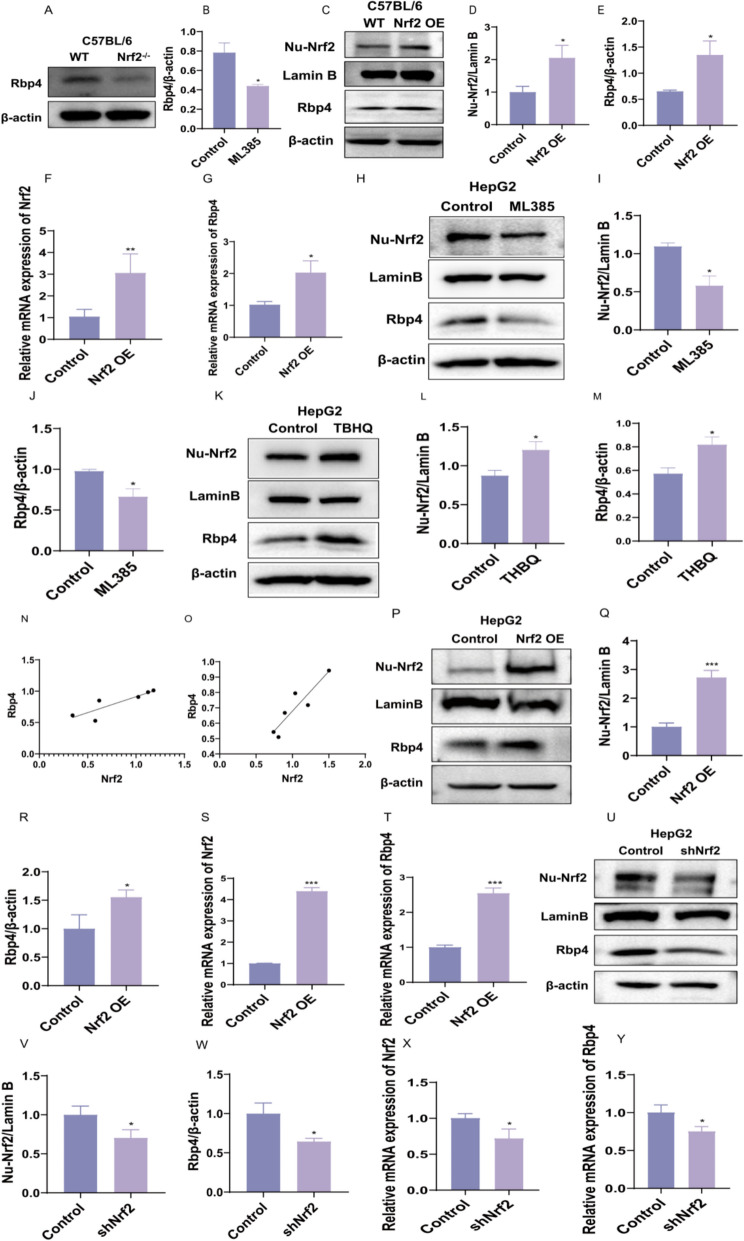
Nrf2 transcriptionally regulates Rbp4 expression in ALD models. **A**, **B** Western blot and quantification of Rbp4 expression in liver tissues of wild-type (WT) and Nrf2 knockout (Nrf2⁻/⁻) mice (n = 5). **C**–**E** Nuclear and cytoplasmic fractionation followed by Western blot showing nuclear Nrf2 (Nu-Nrf2) and Rbp4 expression levels in WT and Nrf2 overexpression (Nrf2 OE) mouse liver tissues. Lamin B and β-actin were used as nuclear and cytoplasmic loading controls, respectively. Quantifications of nuclear Nrf2 (D) and Rbp4 (E) are presented (n = 6). **F**, **G** RT-qPCR detection of Nrf2 and Rbp4 mRNA expression in WT and Nrf2 OE mouse liver tissues (n = 6). **H**–**J** Western blot and quantification of nuclear Nrf2 and Rbp4 in HepG2 cells treated with ML385 (Nrf2 inhibitor) compared to the Control group. Lamin B was used as a nuclear loading control (n = 3). **K**–**M** Western blot of HepG2 cells treated with TBHQ (Nrf2 activator) compared to control cells, showing Nu-Nrf2 and Rbp4 levels. Quantifications of nuclear Nrf2 (**L**) and Rbp4 (**M**) are shown (n = 4). **N**–**O** Correlation analysis between Nrf2 and Rbp4 protein levels, revealing a positive relationship in the tested samples. **P**–**T** Western blot and qPCR analyses of Nrf2 and Rbp4 in HepG2 cells overexpressing Nrf2 (Nrf2 OE) compared to the Control group (n = 3). Nuclear Nrf2 and Rbp4 levels were quantified (Q-R) (n = 3), as well as mRNA expression of Nrf2 (**S**) and Rbp4 (**T**) (n = 3). **U**–**Y** Western blot and RT-qPCR analyses of HepG2 cells with Nrf2 knockdown (shNrf2) compared to control cells. Nuclear Nrf2 and Rbp4 levels (**V**, **W**) and their mRNA expression (**X**, **Y**) were significantly reduced in the knockdown group (n = 3). Compared with the Control group, *P < 0.05, **P < 0.01, ***P < 0.001

First, we validated the relationship between Nrf2 and Rbp4 through both animal models and cell experiments. Protein expression levels of Rbp4 were significantly decreased in the liver of Nrf2^−/−^ mice (Fig. 6A, B). Conversely, adenovirus-mediated Nrf2 overexpression via tail vein injection significantly upregulated Rbp4 mRNA and protein levels (Fig. 6C–G).

In cell experiments, we treated with the Nrf2 inhibitor ML385 and the agonist TBHQ. Western blot and RT-qPCR results showed that the expression of Rbp4 protein and gene decreased significantly after inhibiting Nrf2 nuclear translocation. Upon activation of Nrf2, the expression of Rbp4 was significantly increased, and there was a positive correlation between Nrf2 and Rbp4 expression (Fig. 6H–O).

Overexpression and knockdown of Nrf2 in HepG2 cells were further verified by cell transfection. The results showed that overexpression of Nrf2 increased Rbp4 mRNA expression and protein levels, whereas Nrf2 knockdown reduced Rbp4 expression (Fig. 6P–Y).

**Fig. 7:**
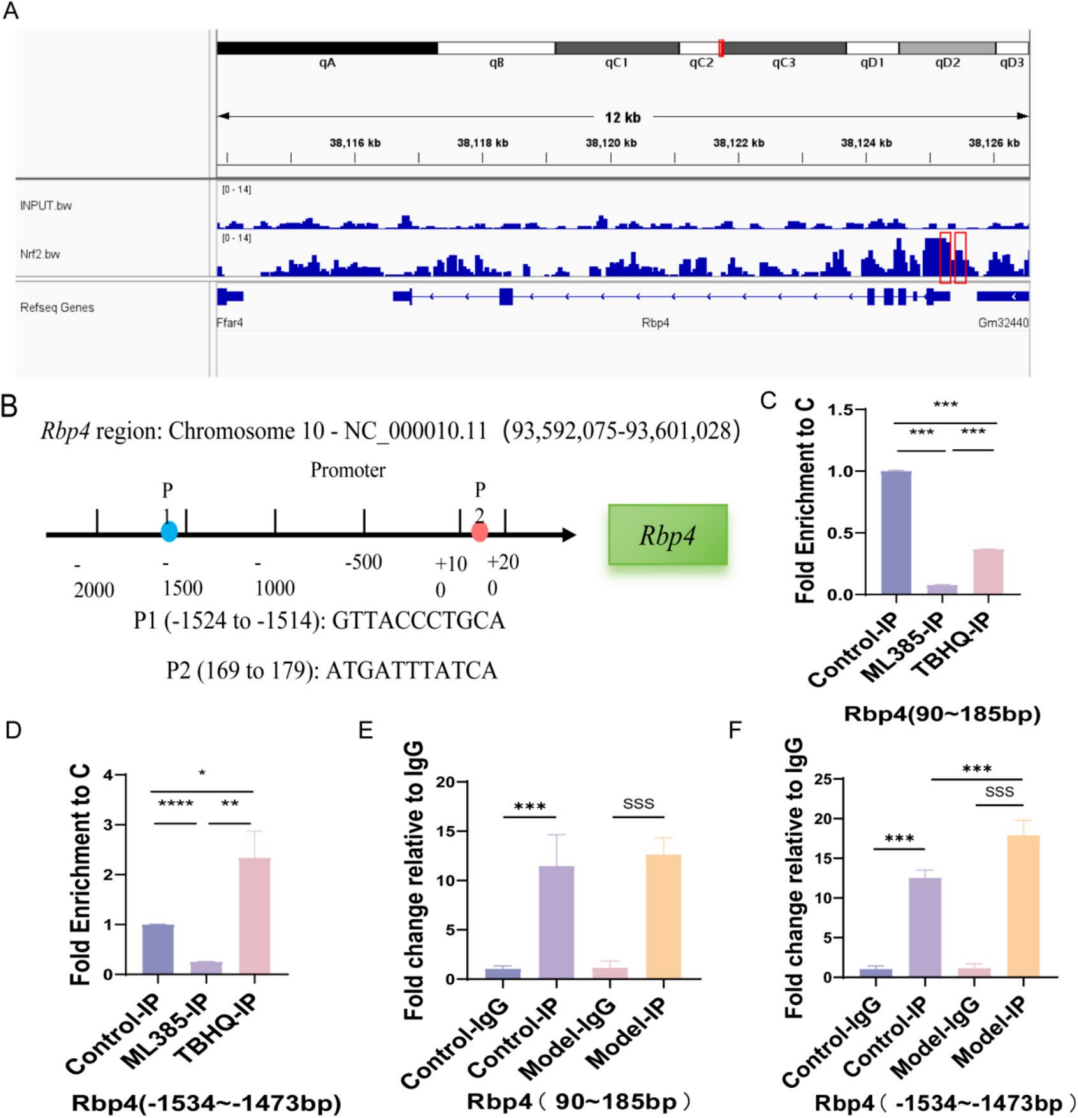
Nrf2 binds to the Rbp4 promoter region and regulates its transcriptional activity. **A** ChIP-seq analysis shows Nrf2 binding sites in the Rbp4 promoter region. **B** Schematic diagram of predicted ARE sequences in the Rbp4 promoter region (− 2000 ~ + 200). **C** ChIP-qPCR results showing Nrf2 binding to the Rbp4 promoter region at 90 ~ 185 bp (n = 3). **D** ChIP-qPCR results showing significant Nrf2 binding at the Rbp4 promoter region − 1534 ~ − 1473 bp (n = 3). **E** ChIP-qPCR results showing significant Nrf2 binding at the Rbp4 promoter region − 1534 ~ − 1473 bp between the normal group and the ALD model group (n = 6). **F** ChIP-qPCR results showing significant Nrf2 binding at the Rbp4 promoter region 90 ~ 185 bp between the normal group and the ALD model group (n = 6). **C**, **D** Compared with the Control group, *P < 0.05, **P < 0.01, ***P < 0.001. **E**, **F** Compared with the Control-IP group, *P < 0.05, **P < 0.01, ***P < 0.001. Compared with the Model-Ig group, ^S^P < 0.05, ^SS^P < 0.01, ^SSS^P < 0.001

Further ChIP-seq analysis, we detected significant Nrf2 binding signals in the Rbp4 promoter region, suggesting that Nrf2 may regulate the expression of Rbp4 through binding to this region (Fig. 7A). Through promoter region sequence analysis, two potential antioxidant response elements (ARE) were predicted: ARE1 (− 1524~− 1514) and ARE2 (+ 169–179) (Fig. 7B). In order to verify the binding strength of Nrf2 to these regions, we conducted ChIP-qPCR experiments, and the results showed that Nrf2 was mainly bound to the Rbp4 promoter − 1534~− 1473 bp region, while the binding was weak in the 90–185 bp region. In addition, Nrf2 agonist TBHQ treatment significantly enhanced the binding of Nrf2 in the promoter region, and the mRNA expression of Rbp4 was significantly up-regulated. Treatment with ML385, an Nrf2 inhibitor, resulted in reduced binding and decreased Rbp4 mRNA levels (Fig. 7C, D). Furthermore, we examined Nrf2 enrichment at the Rbp4 locus in both Control and ALD model groups. ChIP-qPCR analysis revealed statistically significant enrichment in the IP samples within two distinct genomic regions (90–185 bp and − 1534~− 1473 bp) relative to IgG Control group. These findings confirm specific binding interactions between Nrf2 and these regulatory elements of Rbp4. Notably, within the − 1534~− 1473 bp region, we observed a statistically significant increase in Nrf2 binding intensity in the ALD model group compared to Control group. This enhanced interaction suggests that ALD pathogenesis may involve aberrant amplification of Nrf2/Rbp4 axis activity, thereby modulating disease progression through dysregulated target gene expression.

In conclusion, Nrf2 directly promotes transcriptional activation of Rbp4 by binding to the promoter region of Rbp4, resulting in increased expression levels of Rbp4, promoting lipid accumulation and the occurrence and development of liver injury.

## Discussion

**Fig. 8:**
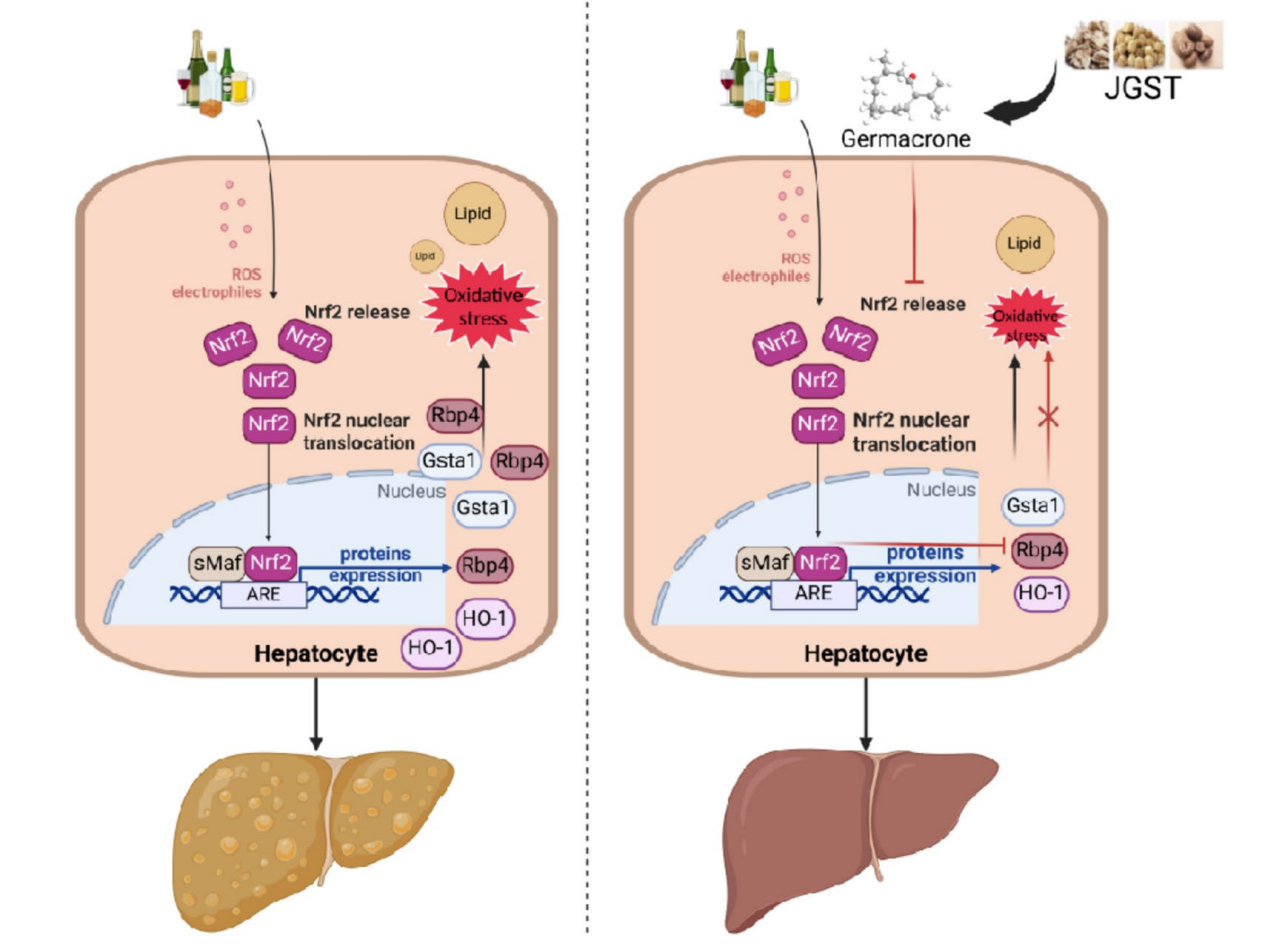
Mechanism of Germacrone in the Treatment of Alcohol-Induced Liver Disease (ALD). Under alcohol exposure, reactive oxygen species (ROS) and foreign electrophilic reagents inhibit the ubiquitination of Nrf2, leading to its nuclear translocation. In the nucleus, Nrf2 binds to antioxidant response elements (AREs), promoting the transcription of downstream target genes, including Rbp4 (lipid transport protein), HO-1, and Gsta1 (oxidative stress-regulated proteins). This process accelerates lipid transport, lipid accumulation, and oxidative damage in hepatocytes, which contributes to the progression of ALD (left panel). In contrast, Germacrone (Germacrone), the active component of Jia-Ga-Song-Tang (JGST), effectively inhibits Nrf2 activation and nuclear translocation, thereby down-regulating the expression of Rbp4, HO-1, and Gsta1. This reduces lipid accumulation and oxidative stress, protecting hepatocytes and improving liver health (right panel)

This study revealed the protective effect of Germacrone on ALD for the first time and clarified the molecular mechanism of Germacrone improvement of hepatic lipid metabolism disorder and oxidative stress damage by regulating the Nrf2/Rbp4 signaling pathway through in-depth studies (Fig. 8). In both in vivo and in vitro models, Germacrone can significantly inhibit the nuclear translocation of Nrf2 and the expression of its downstream lipid transporter Rbp4, thereby effectively alleviating lipid accumulation and oxidative damage in hepatocytes. These results not only identified Rbp4 as a novel downstream effector of Nrf2, but also preliminarily elucidated the regulatory role of this signaling pathway in ALD pathogenesis, providing an important theoretical basis for the potential application of Germacrone in the prevention and treatment of ALD.

ALD is a chronic liver injury caused by long-term excessive alcohol consumption, the core pathological features mainly include oxidative stress imbalance and lipid metabolism disorder. In the process of alcohol metabolism, the production of a large number of reactive oxygen species (ROS) and free radicals will disrupt the balance of the body's antioxidant system and further aggravate liver cell damage and steatosis through lipid peroxidation and inflammatory reactions [8]. In response to oxidative stress, the body activate a series of protective mechanisms to maintain redox balance. As a key regulatory system, the Nrf2 signaling pathway plays a central role in this process by activating antioxidant gene expression, clearing ROS, and maintaining cellular homeostasis [8]. While Nrf2 activation is essential for combating oxidative stress, emerging evidence indicates that its overactivation may contribute to abnormal lipid metabolism. Mechanistically, excessive Nrf2 signaling promotes lipid accumulation and exacerbates liver injury through upregulation of lipid uptake mediators, including the scavenger receptor CD36 and fatty acid transport proteins (FATPs) [10, 17].

In addition to Nrf2, Rbp4 (retinol-binding protein 4) has also been recognized as an important regulator of lipid metabolism disorders in recent years. Existing studies have shown that abnormal Rbp4 expression not only suppresses mitochondrial oxidative metabolism but also exacerbates hepatocyte injury through dual mechanisms involving inflammatory activation and ectopic lipid release [15]. In addition, Emerging evidence further indicates that Rbp4 not only directly participates in lipid metabolism and inflammation regulation within cells but also exerts systemic effects through the exosomal pathway. A pivotal study demonstrated that the exosome Rbp4 was able to significantly enhance lipid accumulation and inflammatory responses in the livers of mice on a high-fat diet by promoting M1 polarization of Kupffer cells (KCs). Mechanistically, the study found that exosome Rbp4 enhances M1-like polarization in Kupffer cells by mediating the NOX2/ROS/NF-κB pathway while activating the JAK2/STAT3 signaling cascade through TNF-α secretion, thereby promoting new adipogenesis in hepatocytes. These findings elucidate the dual regulatory role of Rbp4 in orchestrating the inflammatory microenvironment and metabolic reprogramming, highlighting its pathogenic contribution to non-alcoholic fatty liver disease (NAFLD) progression. This mechanistic insight establishes exosomal Rbp4 as a potential therapeutic target for NAFLD [29]

While previous studies have elucidated the individual roles of Nrf2 and Rbp4 in ALD pathogenesis, their functional interplay remains poorly defined. This study provides the first direct experimental evidence that Nrf2 specifically binds to the Rbp4 promoter region (− 1534 ~ − 1473 bp) and drives its transcriptional activation. This regulatory mechanism was consistently observed in the alcoholic liver disease (ALD) model, with significantly enhanced binding enrichment in the ALD model group compared to Controls. Building upon prior findings, we propose a novel mechanistic paradigm: Nrf2-mediated regulation of Rbp4 expression amplifies the crosstalk between inflammatory signaling and lipid metabolic dysregulation, thereby potentiating hepatic injury. The discovery further refine our understanding of the Nrf2/Rbp4 signaling pathway in lipid metabolism disorder and oxidative stress imbalance and provides a new perspective and molecular target for the study of the pathological mechanism of ALD.

On the basis of exploring the Nrf2/Rbp4 signaling pathway, this study further focuses on the regulatory role of Germacrone in this pathway. Through network pharmacology screening, we identified Germacrone as a core bioactive component of the Tibetan medicinal formulation Jia-ga-song-Tang (JGST). In recent years, Germacrone has gradually become the focus of attention due to its potential in various pharmacological applications such as anticancer, anti-inflammatory, antioxidant, lipid-lowering, antibacterial, insecticidal, and neuroprotective applications [19]. These studies show that Germacrone, as a natural compound, can play a wide range of regulatory roles in a variety of pathological processes, especially in improving oxidative stress imbalances, inhibiting inflammatory responses, and regulating lipid metabolism. Our experimental data demonstrate that Germacrone has a significant hepatoprotective effects against ALD by regulating the Nrf2/Rbp4 signaling pathway to improve liver lipid metabolism disorders and oxidative stress damage. Specifically, Germacrone inhibits Nrf2 nuclear translocation and subsequent Rbp4 transactivation, thereby attenuating lipid accumulation and oxidative damage in hepatocytes.

Combined with the results of this study, the multiple pharmacological effects of Germacrone can be further explained from the molecular mechanism. Firstly, its antioxidant capacity is mediated through modulation of the Nrf2 signaling pathway, effectively counteracting oxidative stress-induced cellular damage. Secondly, the lipid-lowering activity stems from Rbp4 downregulation, which significantly ameliorates hepatic steatosis by reducing lipid droplet accumulation in hepatocytes. This dual-target mechanism not only establishes Germacrone as a promising therapeutic agent for ALD but also extends its potential applicability to other pathologies involving lipid dysmetabolism-oxidative stress crosstalk. Furthermore, Germacrone’s anti-inflammatory properties may synergize with these effects, likely through Rbp4-dependent suppression of Kupffer cell (KC) M1 polarization, thereby reshaping the hepatic inflammatory microenvironment. The precise immunomodulatory pathways warrant systematic investigation in future studies.

It is worth mentioning that the mechanism of action of Germacrone may be consistent with the therapeutic concept of “warm medicine” in Tibetan medical theory. According to the theory, “warm medicine” are postulated to restore the body’s dynamic equilibrium by counteracting pathological states arising from ‘cold’ (grang). The dual regulatory capacity of Germacrone in mitigating oxidative stress and lipid metabolic disorders, as demonstrated in this study, exhibits conceptual congruence with the traditional Tibetan medicine theory that ‘warm medicines’ exert therapeutic effects through homeostatic modulation. While this correlation is grounded in modern pharmacological mechanisms, it provides a scientific framework for reconciling traditional ethnopharmacological wisdom with contemporary biomedical research, thereby advancing the modernization of Tibetan medicinal practices.

In conclusion, combined with the extensive pharmacological effects of Germacrone and the experimental results of this study, its antioxidant, anti-inflammatory, and lipid-lowering properties further expand its application potential in the treatment of alcoholic liver disease and other related diseases and provide an important reference for the modernization of Tibetan medicine and molecular mechanism research.

Although this study revealed that Germacrone may modulate the mechanism of action in ALD by inhibiting the Nrf2/Rbp4 signaling pathway, several limitations remain. First, the model selection is simplistic: this study employed an animal model of ALD induced solely by alcohol, without including a combined model involving both a high-fat diet and alcohol. Future studies should incorporate more complex pathological models to comprehensively assess the therapeutic potential of Germacrone. Second, the current study lacks in-depth discussion of the mechanism. On one hand, based on the tissue-specific role of the Nrf2/Rbp4 pathway in hepatocytes, the absence of liver-conditional knockout validation in current in vivo experiments limits mechanistic confirmation. Furthermore, although existing data suggest the critical involvement of this signaling pathway in alcoholic liver disease (ALD), the current dataset requires further expansion and refinement. Therefore, future studies will focus on utilizing liver-specific knockout mouse models to investigate the functional mechanisms of this pathway, while validating its pivotal role in ALD progression through complementary in vitro and in vivo experiments and reverse ChIP-qPCR experiments. These efforts aim to more precisely elucidate its tissue-specific regulatory roles in ALD pathogenesis. On the other hand, while this study focuses on the Nrf2/Rbp4 signaling pathway, it does not explore whether Germacrone synergistically modulates oxidative stress and lipid metabolism through other signaling pathways or molecular targets. The multi-target regulatory mechanism of Germacrone can be further revealed by proteomics and metabolomics in the future. In addition, more importantly, the current results lack clinical translational validation. Although Germacrone has shown good therapeutic effect in in vivo and in vitro models, its efficacy and safety in the clinic still need to be further verified, especially the optimization of drug dosage and metabolic pathway.

## Conclusion

To summarize, this study revealed the molecular mechanism of Germacrone in improving oxidative stress and lipid metabolism disorder in ALD through inhibition of the Nrf2/Rbp4 pathway, using integrated in vivo and in vitro experimental systems. Furthermore, we demonstrated that Nrf2 directly binds to the Rbp4 promoter region to regulate its expression, thereby highlighting the critical role of the Nrf2/Rbp4 pathway in ALD pathogenesis. These findings provide a foundational theoretical basis for developing Germacrone as a novel therapeutic candidate for ALD.

## Supplementary Information

Supplementary Material 1. Figure 1 Proteomic quality control analysis.Peptide length profile;peptide matching error distribution map;peptide quantity distribution map

Supplementary Material 2. Figure 2. Network pharmacological analysis of JGST on ALD.The common targets of JGST and ALD were screened by Venny diagram analysis;Visualization of overlapping targets between JGST and ALD;The top 25 core targets network diagram

Supplementary Material 3. Figure 3. KEGG Enrichment Pathway Diagram

Supplementary Material 4. Figure 4.FASN, SREBP1, SCD mRNA expression in HepG2 cells in the Control group, Model group, low-, medium-, and high-dose Germacrone groups was analyzed by RT-qPCR

Supplementary Material 5. Table 1. Table of component-target numbers

Supplementary Material 6. Table 2. Target corresponding component importance map

Supplementary Material 7. Table 3. The corresponding target between JGST and ALD

## Data Availability

All data generated in the present study may be requested from the corresponding authors.

## Abbreviations

ALD: Alcohol-related liver disease
JGST: Jia-Ga-Song-Tang
Nrf2: Nuclear Factor erythroid 2-Related Factor 2;
Rbp4: Retinol binding protein 4
HO-1: Heme oxygenase-1
Gsta1: Glutathione *S*-transferase alpha 1
NQO1: NAD(P)H quinone dehydrogenase 1
FASN: Fatty acid synthase
SREBP1-C: Sterol Regulatory Element-Binding Protein 1 Cleaved
SCD: Stearoyl-CoA desaturase
ALT: Alanine aminotransferase
AST: Aspartate aminotransferase
TG: Triglycerides
GSH: Glutathione
MDA: Malondialdehyde
SOD: Superoxide dismutase
LDL-C: Low density lipoprotein
HDL-C: High density lipoprotein cholesterol
