# Supplementary figures and images for: The effect and mechanism of Germacrone in ameliorating alcoholic fatty liver by inhibiting Nrf2/Rbp4
Source: Chin Med. 2025 May 29;20:77. doi: 10.1186/s13020-025-01132-y (PMC12121245; doi:10.1186/s13020-025-01132-y)

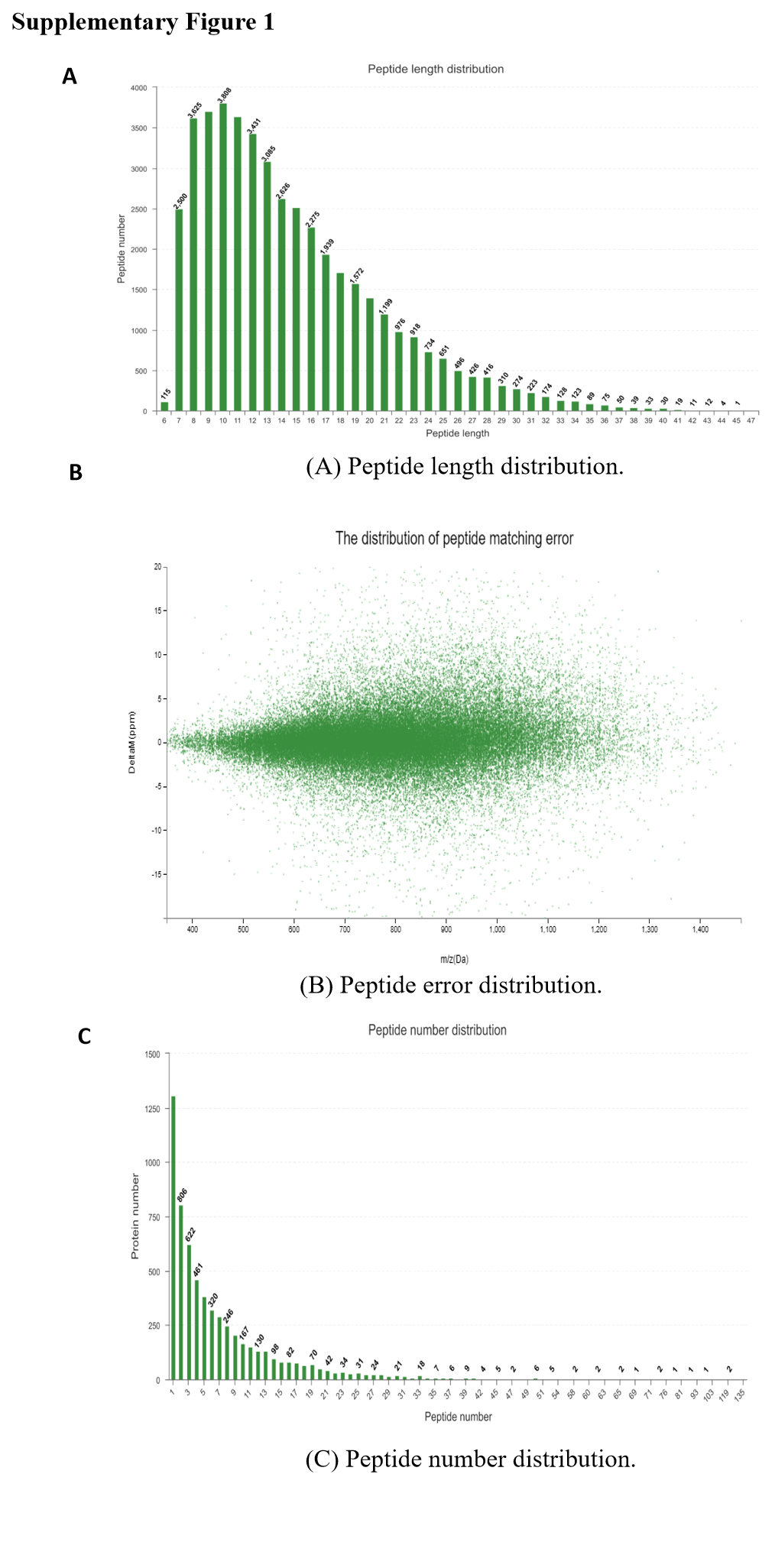

Supplement: Supplementary file 1 — Supplementary Material 1. Figure 1 Proteomic quality control analysis.Peptide length profile;peptide matching error distribution map;peptide quantity distribution map [file 13020_2025_1132_MOESM1_ESM.tif]

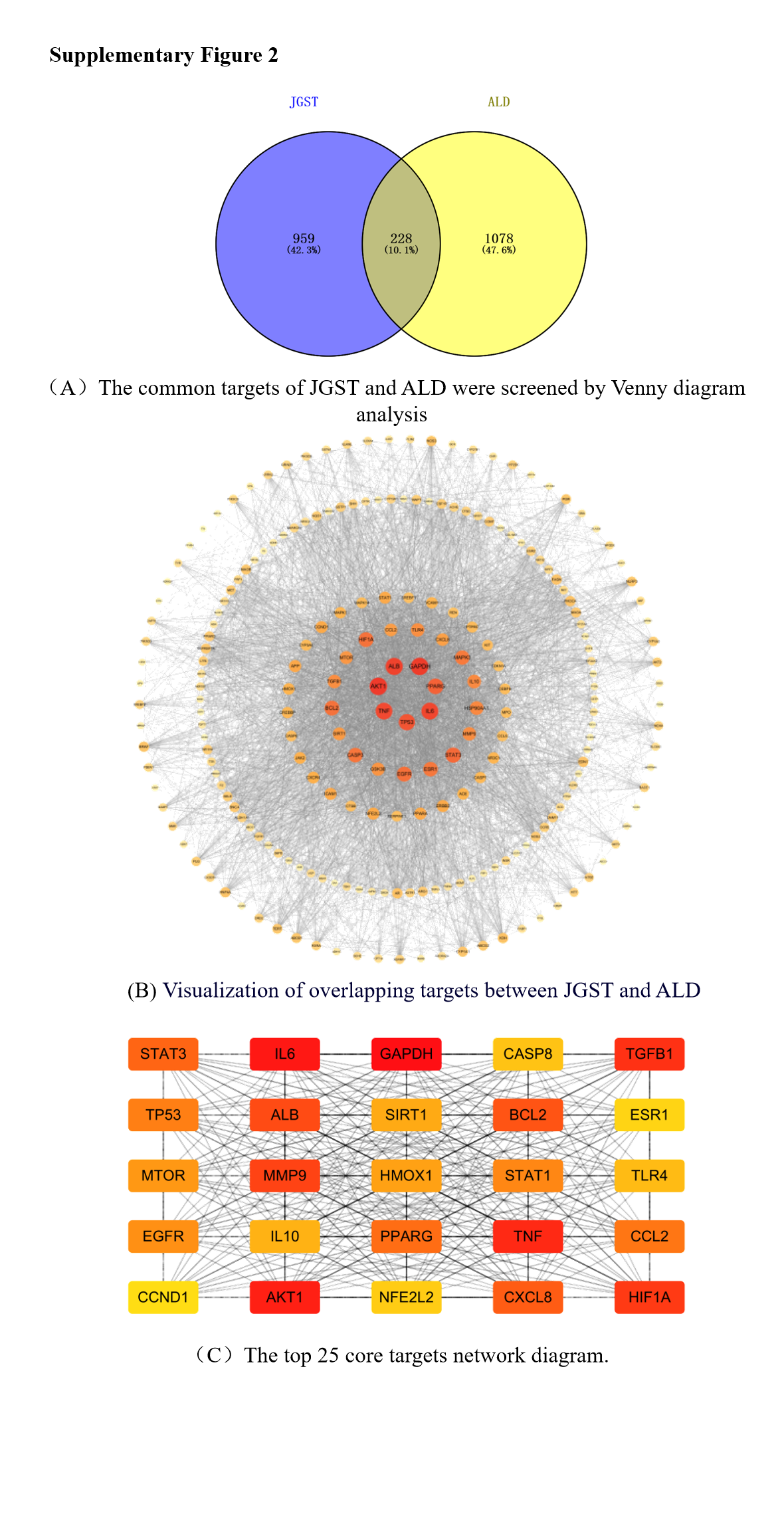

Supplement: Supplementary file 2 — Supplementary Material 2. Figure 2. Network pharmacological analysis of JGST on ALD.The common targets of JGST and ALD were screened by Venny diagram analysis;Visualization of overlapping targets between JGST and ALD;The top 25 core targets network diagram [file 13020_2025_1132_MOESM2_ESM.tif]

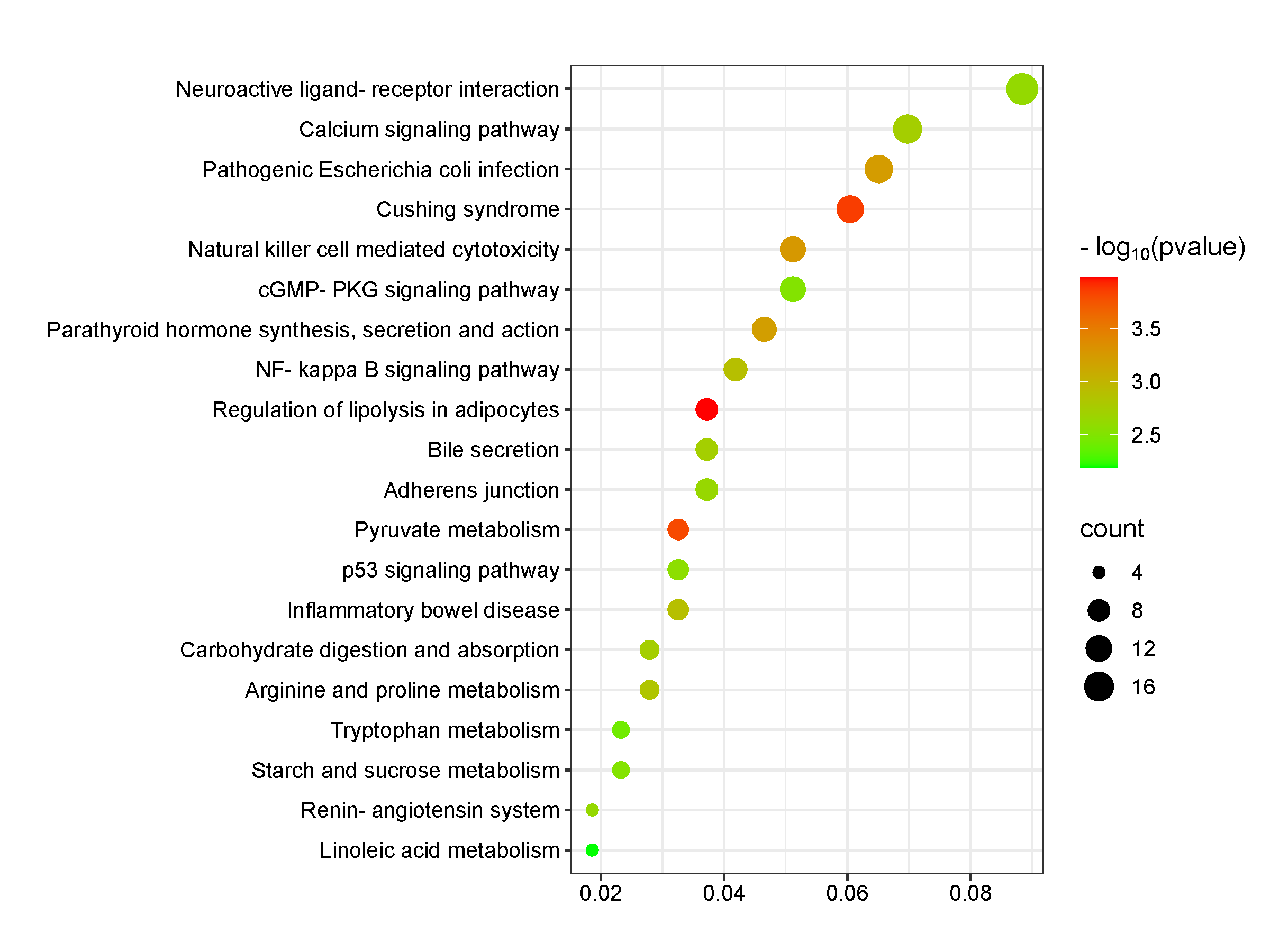

Supplement: Supplementary file 3 — Supplementary Material 3. Figure 3. KEGG Enrichment Pathway Diagram [file 13020_2025_1132_MOESM3_ESM.tif]

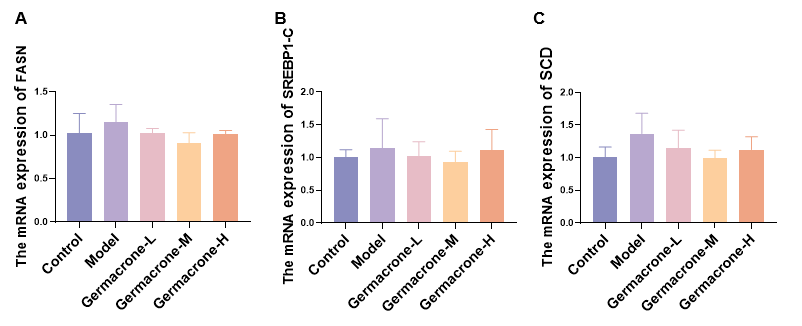

Supplement: Supplementary file 4 — Supplementary Material 4. Figure 4.FASN, SREBP1, SCD mRNA expression in HepG2 cells in the Control group, Model group, low-, medium-, and high-dose Germacrone groups was analyzed by RT-qPCR [file 13020_2025_1132_MOESM4_ESM.tif]
